# ABL-N-induced apoptosis in human breast cancer cells is partially mediated by c-Jun NH_2_-terminal kinase activation

**DOI:** 10.1186/bcr2475

**Published:** 2010-01-25

**Authors:** Bin Liu, Mei Han, Rong-Hua Sun, Jun-Jie Wang, Yan-Ping Zhang, Di-Qun Zhang, Jin-Kun Wen

**Affiliations:** 1 Department of Biochemistry and Molecular Biology, Institute of Basic Medicine, Hebei Medical University, No.361, Zhongshan East Road, Shijiazhuang, 050017, China; 2 College of Pharmacy, Hebei Medical University, No.361, Zhongshan East Road, Shijiazhuang, 050017, China

## Abstract

**Introduction:**

The present study was designed to determine the possibility of acetylbritannilactone (ABL) derivative 5-(5-(ethylperoxy)pentan-2-yl)-6-methyl-3-methylene-2-oxo-2,3,3a,4,7,7a-hexahydrobenzofuran-4-yl 2-(6-methoxynaphthalen-2-yl)propanoate (ABL-N) as a novel therapeutic agent in human breast cancers.

**Methods:**

We investigated the effects of ABL-N on the induction of apoptosis in human breast cancer cells and further examined the underlying mechanisms. Moreover, tumor growth inhibition of ABL-N was done in xenograft models.

**Results:**

ABL-N induced the activation of caspase-3 in estrogen receptor (ER)-negative cell lines MDA-MB-231 and MDA-MB-468, as evidenced by the cleavage of endogenous substrate Poly (ADP-ribose) polymerase (PARP). Pretreatment of cells with pan-caspase inhibitor z-VAD-fmk or caspase-3-specific inhibitor z-DEVD-fmk inhibited ABL-N-induced apoptosis. ABL-N treatment also resulted in an increase in the expression of pro-apoptotic members (Bax and Bad) with a concomitant decrease in Bcl-2. Furthermore, c-Jun-NH_2_-terminal kinase (JNK) and p38 mitogen-activated protein (MAP) kinase (p38) were activated in the apoptosis induced by ABL-N and JNK-specific inhibitor SP600125 and JNK small interfering RNA (siRNA) antagonized ABL-N-mediated apoptosis. However, the p38-specific inhibitor SB203580 had no effect upon these processes. Moreover, neither of the caspase inhibitors prevented ABL-N-induced JNK activation, indicating that JNK is upstream of caspases in ABL-N-initiated apoptosis. Additionally, in a nude mice xenograft experiment, ABL-N significantly inhibited the tumor growth of MDA-MB-231 cells.

**Conclusions:**

ABL-N induces apoptosis in breast cancer cells through the activation of caspases and JNK signaling pathways. Moreover, ABL-N treatment causes a significant inhibition of tumor growth *in vivo*. Therefore, it is thought that ABL-N might be a potential drug for use in breast cancer prevention and intervention.

## Introduction

Breast cancer is one of the most common cancers among women in both developed and underdeveloped countries. It is the malignancy with the highest incidence and death rate for women [1,2]. However, the efficacy of the present drugs is very limited, and it is urgent to find the anticancer compounds that can target multiple points in the apoptotic cascade to achieve synergistic actions. Chinese herbs have obtained considerable attention for the prevention and treatment of certain cancer types in clinical studies [3-6]. In many cases, the extracts obtained from the plants are not highly effective and require chemical modification for improved potency and toxicity profile [7-9]. Thus, studies of naturally plant-based agents could supply new strategies for the management of cancer and related diseases [7,10,11].

Recently, several phytochemicals that have been used in clinical cancer chemotherapy were derived from herbs and plants, such as paclitaxel [5,12], etoposide [13], camptothecin [4] and vinca alkaloids [14]. Acetylbritannilactone (ABL) is a sesquiterpene lactone abundant in *Inula britannica L*, which is used to treat bronchitis and inflammation. In the previous work, it is demonstrated that ABL inhibits the expression of inflammation-associated genes and it possesses anticancer properties [15-19]. In the course of our continuing search for cytotoxic ABL analogues, we synthesized the compound 5-(5-(ethylperoxy)pentan-2-yl)-6-methyl-3-methylene-2-oxo-2,3,3a,4,7,7a-hexahydrobenzofuran-4-yl 2-(6-methoxynaphthalen-2-yl)propanoate (ABL-N), which in preliminary studies showed exceptional anti-proliferative activity against several human cancer cell types. Here, we showed that ABL-N was more potent than ABL in the ability to induce apoptosis, at a low concentration, of human breast cancer cells and investigated the therapeutic potential of the ABL-N and its underlying mechanism of action.

## Materials and methods

### Preparation of ABL and ABL-N

**Figure 1:**
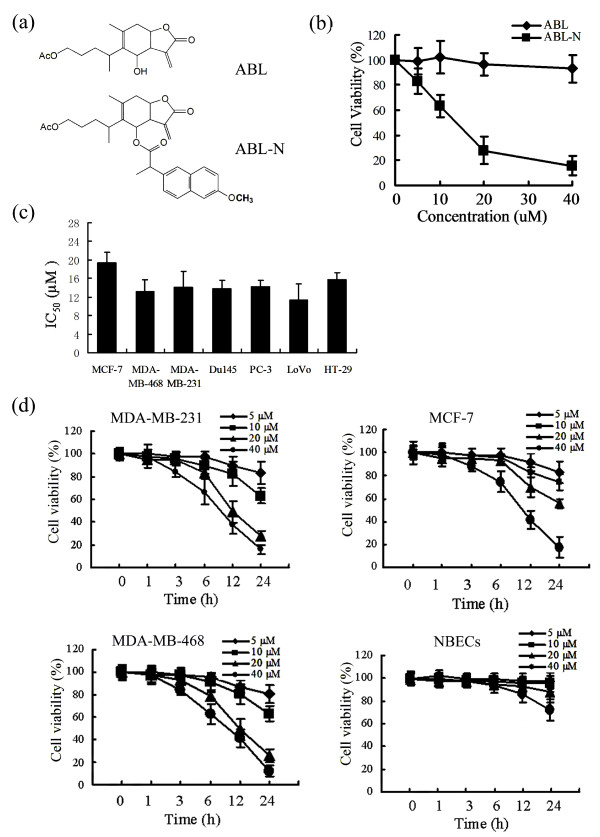
**Effect of ABL and ABL-N on cancer cell lines**. **(a) **The chemical structures of ABL and ABL-N. **(b) **The differences of growth inhibition activity between ABL and ABL-N in MDA-MB-231 cells. **(c) **Effects of ABL-N on the viability of various cancer cell lines. Cells were treated with ABL-N for 24 hours and cell viability was determined by the MTT assay. The IC_50 _is the concentration of ABL-N that reduced the cell viability by 50% under the experimental conditions (n = 6). **(d) **Time- and dose-dependent inhibition of cell viability by ABL-N treatment in MDA-MB-231, MCF-7, MDA-MB-468 and NBECs. Cells were treated with 5, 10, 20 or 40 μM ABL-N for 1, 3, 6, 12 and 24 hours and cell viability was assessed by MTT assay (n = 6). Results represent the means ± SE from three independent experiments.

Silica gel column chromatography was used to isolate ABL from *Inula britannica L *grown in Shan-xi Province in China. ABL-N was synthesized to improve efficacy and pharmacologic characteristics by substitution at C-6 of ABL (Figure 1a). These compounds were characterized by nuclear magnetic resonance and mass spectroscopy. The purified ABL and ABL-N were dissolved in ethanol at 1,000-fold final concentration and added to cells in exponential growth. The effects of ABL and ABL-N on our experiments were compared with the same concentration of ethanol as vehicle.

### Reagents

3-(4,5-dimethylthiazol-2-yl)-2,5-diphenyltetrazoliumbromide (MTT), DMSO, 4,6-diamidino-2-phenylindole (DAPI), small interfering RNA (siRNA) specific for human JNK mRNA and control siRNA were obtained from Sigma Chemicals (St. Louis, MO, USA). LipofectAMINE 2000, Dulbecco's modified Eagle's medium (DMEM), penicillin, and streptomycin were purchased from Invitrogen (Carlsbad, CA, USA). The antibodies specific for Poly (ADP-ribose) polymerase (PARP), c-Jun NH_2_-terminal kinase (JNK), phospho-JNK, p38 MAP kinase (p38) and phospho-p38 were obtained from Cell Signaling Technology (Beverly, MA, USA). Antibodies against extracellular signal-regulated kinase (ERK), phospho-ERK, caspase-3, caspase-9, c-Jun, phospho-c-Jun, Bcl-2, Bax, Bad and secondary antibodies were from Santa Cruz Biotechnology (Santa Cruz, CA, USA). Pan-caspase inhibitor (z-VAD-fmk) was from Promega (Madison, WI, USA) and caspase-3-specific inhibitor (z-DEVD-fmk) was obtained from CalBiochem (San Diego, CA, USA). Unless otherwise indicated, all other reagents used in this study were obtained from Sigma Chemicals.

### Cell lines and culture conditions

The human breast cancer cell lines (MCF-7, MDA-MB-468 and MDA-MB-231), the human prostate carcinoma cells (Du145 and PC-3), and the human colon carcinoma cells (LoVo and HT-29) were from the American Type Culture Collection. Normal human breast epithelial cells (NBECs) were cultured and characterized as described previously from reduction mammoplasty specimens [20,21]. Cells were grown in a 5% CO_2 _atmosphere at 37°C in DMEM supplemented with 100 units/ml penicillin, 100 μg/ml streptomycin, 1% nonessential amino acids, and 10% fetal bovine serum (v/v). All treatments were carried out on cells at 60 to 80% confluence.

### Cell viability assay

Loss of cell viability was measured by the MTT assay. Cells were seeded at 1 × 10^4 ^cells/well in 96-well plates and allowed to grow in the growth medium for 24 hours. Cells were then treated with indicated concentrations of ABL-N for various time periods. After drug treatment, cells were incubated with 5 mg/ml MTT for two hours, and subsequently solubilized in DMSO. The absorbency at 570 nm was then measured using an enzyme-linked immunosorbent assay (ELISA) reader. The IC_50 _is the concentration agent that reduced the cell viability by 50% under experimental conditions. Experiments were repeated at least three times, and the data were expressed as the means ± SE.

### Nuclear staining assay

After treatment, the cells were harvested, washed with phosphate-buffered saline (PBS), and fixed in 70% ethanol for 30 minutes. The fixed cells were placed on slides and stained with 1 mg/ml DAPI for 15 minutes. Excess dye was washed off with PBS. Nuclear morphology was observed via a fluorescence microscope.

### Cell cycle analysis

Cell cycle distribution was determined using flow cytometry analysis. Briefly, after treatment, cells were harvested with 0.25% trypsin, washed in PBS and centrifuged. The cells were fixed in ice-cold 75% ethanol for at least 30 minutes. Cells were washed and resuspended in PBS containing 25 μg/ml RNase and 0.5% Triton X-100. Samples were then incubated with 50 μg/ml propidium iodide (PI) at 37°C for 30 minutes and analyzed in a flow cytometer (Becton Dickinson, San Jose, CA, USA).

### Cell apoptosis assay

Cell apoptosis was measured by ELISA and flow cytometry, respectively. For ELISA, the cells seeded in 96-well plates (1 × 10^4 ^cells/well) were treated with ABL-N at 5, 10, 20 and 40 μM for 24 hours. Both floating and adherent cells were collected and lysed. Each concentration of ABL-N was repeated five times. The induction of apoptosis by the agent was evaluated with a Cell Death Detection ELISA^Plus ^kit (Roche Diagnostics, Mannheim, Germany) according to the manufacturer's instruction. Photometric enzyme immunoassay was used to quantitatively determine the formation of cytoplasmic histone-associated DNA fragments in the form of mononucleosomes and oligonucleosomes after apoptosis of the cells [22]. Measurements were made using an ELISA reader at 405 nm and the results were calculated as the ratio of the absorbance of the ABL-N-treated cells/absorbance of untreated cells.

For flow cytometry, briefly, after treatment, cells were collected and stained with Annexin V and PI staining using annexin V-FITC apoptosis kit (BD Biosciences, San Diego, CA, USA) according to the manufacturer's instruction. Early apoptotic (annexin V-positive and PI-negative) cells were distinguished from late apoptotic (annexin V and PI double-positive) or necrotic (PI-positive) cells by a flow cytometric analysis.

### Western blot analysis

The cellular lysates were subjected to Western blot analysis as described previously [16,23]. Scanned images were quantified using TotalLab TL120 software (Nonlinear Dynamics Ltd., Newscastle, United Kingdom).

### JNK activity assay

Kinase activity of JNK was assayed with a nonradioactive assay kit according to enclosed manufacturer's procedures of Cell Signaling Technology. Briefly, after the cells were treated, the lysates were prepared using a lysis buffer (20 mM Tris (pH 7.5) containing 150 mM NaCl, 1 mM EDTA, 1 mM EGTA, 1% Triton, 2.5 mM sodium pyrophosphate, 1 mM β-glycerolphosphate, 1 mM phenylmethylsulfonyl fluoride, 1 mM Na_3_VO_4_, and 1 μg/ml leupeptin). JNK in approximately 250 μg of proteins in each sample lysate was pulled down selectively by an N-terminal c-Jun (residues 1 to 89) fusion protein that were bound to glutathione sepharose beads at 4°C overnight with gentle rocking. Thereafter beads were washed twice with the lysis buffer and twice with kinase buffer (25 mM Tris (pH 7.5) containing 5 mM β-glycerolphosphate, 2 mM dithiothreitol, 0.1 mM sodium orthovanadate, and 10 mM MgCl_2_). After the washings, pellets were suspended in 50 μl of kinase buffer supplemented with 200 μM ATP and incubated for 30 minutes at 30°C, during which c-Jun fusion proteins were phosphorylated by the activated JNK. JNK activity was analyzed by Western blotting using a specific phospho-c-Jun (Ser63) antibody. To determine the direct effect of ABL-N on JNK activity, *in vitro *cell-free kinase assays were also performed using purified recombinant GST-JNK1 fusion proteins (SignalChem, Richmond, British Columbia, Canada). ABL-N and purified GST-JNK1 fusion proteins were incubated for 12 hours and JNK activity assay was also performed in a similar manner.

### Measurement of caspase activities

Cells were treated with ABL-N, and the caspase-2, caspase-3/7, caspase-6, caspase-8, and caspase-9 activities in the cleared lysates were measured by using Caspase-Glo 2, Caspase-Glo 3/7, Caspase-Glo 6, Caspase-Glo 8, and Caspase-Glo 9 assays (Promega) according to the manufacturer's protocols. Luminescence was quantified using an ELISA reader. Blank values were subtracted, and increases in caspase activities were expressed as fold increase and calculated based on activities measured from untreated cells. Each sample was measured in triplicates.

### Transfection of siRNA

siRNAs specific for JNK (JNK siRNA) and control siRNA were transiently transfected into cells using transfection reagent (Lipofectamine 2000; Invitrogen) according to the manufacturer's protocol. JNK protein levels were analyzed by Western blotting to confirm adequate silencing of the genes at 48 hours.

### Human breast tumor xenograft experiments

The animal study was performed via a protocol approved by the governmental committee for animal research. Female BALB/c nude mice (four- to five-weeks old) were injected s.c. with MDA-MB-231 cells (6 × 10^6^) per mouse at both flanks. After six days, when tumors reached a size of about 100 mm^3^, mice were randomly grouped and treated by daily i.p. injection with ABL-N at concentrations of 15 mg/kg body weight, or vehicle (10% DMSO, 10% ethanol in water). Mice were weighed at least twice a week to assess toxicity of the treatment, and the tumor size was measured every other day using calipers and their volumes were calculated according to a standard formula: width^2 ^× length/2. Mice were sacrificed after 34 days of treatment when control tumors reached about 1,600 mm^3^.

### Statistical analysis

Normal distribution of data was verified using the Shapiro-Wilk test. Statistical analysis was performed with the two-tailed, unpaired Student's *t*-test using SPSS 11.0 software. Results are given as means ± SE. A value of *P <*0.05 was considered statistically significant.

## Results

### ABL-N reduces the viability of various carcinoma cell lines

The inhibitory effects of ABL and ABL-N on various carcinoma cell lines were estimated using the MTT cellular survival assay. MDA-MB-231 cells were treated for 24 hours with various concentrations of ABL and ABL-N, and cell viability was expressed as percentage of untreated cells. Figure 1b showed that ABL did not affect cell viability at up to 40 μM; whereas this same concentration of ABL-N inhibited cell growth by 85%. It indicated that ABL-N was dramatically more effective than ABL in inhibiting the growth of cells.

Next, we examined the effects of ABL-N on the viability of other human carcinoma cell lines. The results showed that ABL-N treatment inhibited cell growth with similar IC_50 _(approximately 12 to 20 μM) after a 24-hour treatment (Figure 1c). It indicated that ABL-N was a broad-spectrum inhibitory agent of the human carcinoma cells.

Furthermore, the sensitivity of three human breast cancer cells to ABL-N was assessed. These cells were chosen because they represent estrogen receptor (ER)-negative (MDA-MB-231 and MDA-MB-468) and ER-positive (MCF-7) cell lines. The data showed that the inhibitory effects of ABL-N on cell viability occurred very fast. Treating MDA-MB-231 cells for 12 hours with 20 and 40 μM ABL-N reduced cell viability by 51% and 62%, respectively (Figure 1d). Similar results were observed in MDA-MB-468 and MCF-7 cells (Figure 1d). These results showed that there was no relationship between estrogen receptor status and cytotoxic effects of ABL-N.

Moreover, we assessed whether ABL-N had any differential sensitivity to normal versus cancer cells. As shown in Figure 1d, we found that the sensitivity of the NBECs to ABL-N was much lower, with ABL-N only having a significant effect on the viability (28% reduction, *P <*0.05) of the NBECs following 24 hours of treatment at the 40 μM dose, suggesting that ABL-N may have potential selectivity toward tumor cells.

### ABL-N arrests cells in G_2_/M phase of the cell cycle

**Figure 2:**
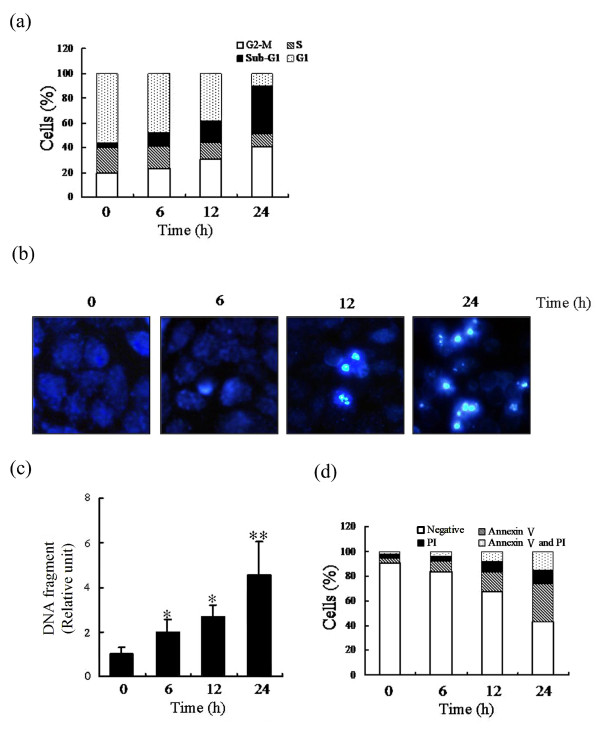
**Effect of ABL-N on cell cycle progression and apoptosis in breast cancer cells**. After treatment with 20 μM ABL-N for indicated times, MDA-MB-231 cells were harvested. **(a) **The percentage of cell cycle distribution for cells done in triplicate with similar results. **(b) **Nuclear condensation was shown by DAPI-staining assay (magnification, ×100). **(c) **DNA fragmentation was evaluated using a Cell Death Detection ELISA^Plus ^kit. The data are expressed as means ± SE of three separate experiments.*P <0.05, **P <0.01, as compared with the group without ABL-N treatment. **(d) **The apoptotic status was determined by Annexin V/PI staining method. Percentages of negative (viable) cells, annexin V-positive (early apoptotic) cells, PI-positive (necrotic) cells, or annexin V and PI double-positive (late apoptotic) cells were shown (mean of three independent experiments) by a flow cytometric analysis.

Because ABL-N can effectively inhibit cell viability, we reasoned that this effect might be attributable to its ability to interfere with the cell cycle. MDA-MB-231 cells were incubated with 20 μM ABL-N for 6, 12 and 24 hours, and the cell cycle analysis was done by PI uptake. Figure 2a shows that the ratio of cells in G_2_/M phase and the cells with hypodiploid DNA contents (sub-G_1_) were significantly accumulated over the treatment periods. In contrast, the cell cycle profile did not change over a 24-hour period in cells treated with the vehicle only (data not shown). Moreover, we also analyzed cell cycle in MDA-MB-468 and MCF-7 cells and found the G_2_/M arrest induced by ABL-N as well (data not shown). Thus, it suggested that the inhibitory effect of ABL-N on breast cancer cells might be, at least in part, due to a G_2_/M arrest of the cell cycle.

### ABL-N induces apoptosis in breast cancer cells

We next analyzed whether the ABL-N-induced cell viability reduction in human breast cancer cells involved apoptosis. MDA-MB-231 cells were treated with 20 μM ABL-N and apoptosis was assayed by two different methods. DAPI staining showed that the condensed and fragmented nuclei increased with ABL-N treatment (Figure 2b). Nucleosome fragmentation (an indicator of apoptosis) further determined by Cell Death Detection ELISA^PLUS ^confirmed that cells treated for six hours with 20 μM ABL-N underwent apoptosis, with the highest percentage of apoptotic cells seen at 24 hours (Figure 2c). In addition, an annexin V-binding assay showed that ABL-N treatment induced apoptosis but not necrosis in MDA-MB-231 cells (Figure 2d). We also obtained similar results when MDA-MB-468 and MCF-7 cells were treated with ABL-N (data not shown).

### ABL-N induces the activities of caspase in breast cancer cells

**Figure 3:**
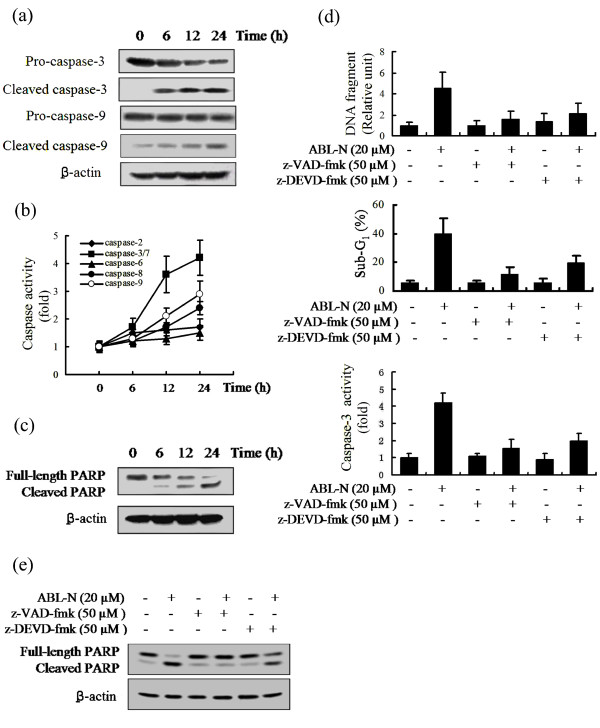
**Induction of caspase activities by ABL-N**. MDA-MB-231 cells were treated with 20 μM ABL-N for indicated times. **(a) **Whole cell protein lysates were prepared and subjected to Western blot analysis for detection of cleavage of caspase-3 and caspase-9. **(b) **Induction of caspase activities by ABL-N in MDA-MB-231 cells. **(c) **Cleavage of PARP was induced by ABL-N. **(d) **Cells were pretreated with 50 μM of either z-VAD-fmk or z-DEVD-fmk for one hour, followed by 20 μM ABL-N for 24 hours, and caspase-3 activity, DNA fragmentation and sub-G_1 _DNA contents were determined. The data are expressed as means ± SE of three separate experiments. **(e) **Abrogation of PARP cleavage by caspase inhibitors.

To investigate the activation of caspases in ABL-N-induced apoptosis, the proteolytic activation of caspase-3 and caspase-9 was examined. As shown in Figure 3a, ABL-N (20 μM) treatment resulted in a significant increase in the active form of cleaved caspase-3 and cleaved caspase-9 in MDA-MB-231 cells. The cleavage products were detectable as early as six hours after exposure of cells to ABL-N. Furthermore, caspase activities were measured with Caspase-Glo assays. As shown in Figure 3b, treatment with ABL-N induced the activation of caspases-3/7, -8 and -9 in MDA-MB-231 cells. Under similar conditions, ABL-N also stimulated caspase-2 and -6 activities in a lesser extent. Similar results were found in MDA-MB-468 cells (data not shown). However, in ER-positive cell MCF-7, the activities of caspase-3/7 and -9 were not affected by ABL-N, although caspase-2 and -6 were activated to a level similar to that seen in MDA-MB-231 and MDA-MB-468 cells (data not shown). However, we found that caspase-8 activity is significantly higher in MCF-7 cells treated with ABL-N, with the activity being increased by 2.8-fold of the untreated cells at 24 hours. The activation of caspases in MDA-MB-231 cells was further confirmed by detecting the degradation of PARP, which is an endogenous substrate of activated caspase-3 and its cleavage is considered a hallmark of apoptosis [24-26]. Treatment of MDA-MB-231 cells with ABL-N resulted in cleavage of PARP to an 85 kDa fragment (Figure 3c).

### Caspase inhibitors attenuate the induction of apoptosis by ABL-N

To define the role of caspase activation in ABL-N-induced apoptosis, we treated MDA-MB-231 cells with pan-caspase inhibitor z-VAD-fmk (50 μM) and caspase-3-specific inhibitor (z-DEVD-fmk) (50 μM) before challenge with ABL-N (20 μM). z-VAD-fmk pretreatment for one hour abrogated ABL-N-induced apoptosis as measured by the nucleosome fragmentation and the appearance of sub-G_1 _cells (Figure 3d). Similar results were observed in both MDA-MB-468 and MCF-7 cells (data not shown). The caspase-3-specific inhibitor z-DEVD-fmk also reduced ABL-N-induced apoptosis in MDA-MB-231 cells (Figure 3d). Moreover, both of the caspase inhibitors abolished caspase-3 activity in MDA-MB-231 cells (Figure 3d). In addition, the cleavage of PARP was attenuated by pretreatment of cells with z-VAD-fmk or with z-DEVD-fmk in MDA-MB-231 cells (Figure 3e). These results suggested that activation of a caspase cascade was essential for ABL-N-induced apoptosis in breast cancer cells.

### ABL-N modulates the expression of Bcl-2 family proteins in breast cancer cells

**Figure 4:**
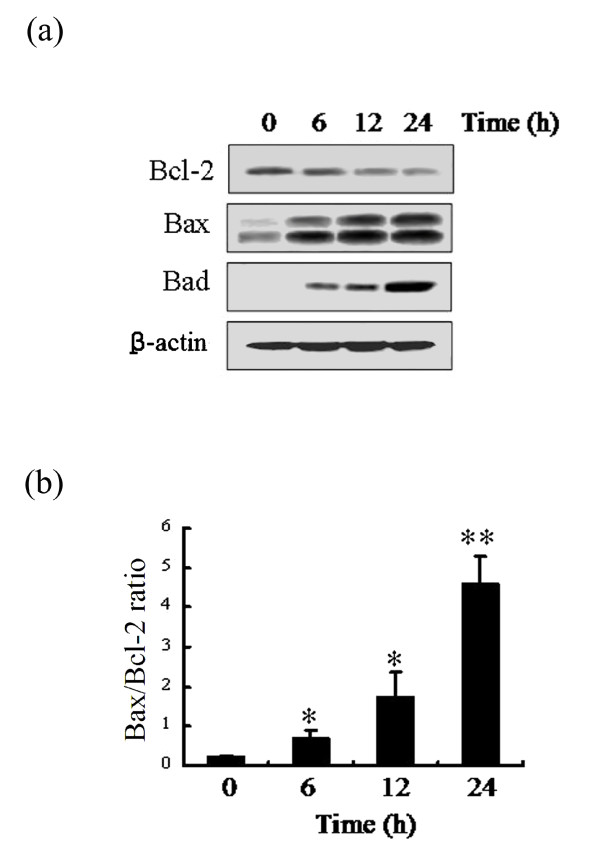
**Effects of ABL-N on the expression of Bcl-2 family**. **(a) **Treatment of ABL-N decreased the level of the anti-apoptotic protein Bcl-2, but increased the expression of the pro-apoptotic proteins Bax and Bad in MDA-MB-231 cells. **(b) **Effect of ABL-N on the Bax/Bcl-2 ratio in MDA-MB-231 cells. The data obtained from the Western blot analysis of Bax and Bcl-2 were used to evaluate the effect of ABL-N on the Bax/Bcl-2 ratio. The densitometric analysis of Bax and Bcl-2 bands was performed using TotalLab TL120 software, and the data (relative density normalized to β-actin) were plotted as Bax/Bcl-2 ratio. Data are expressed as means ± SE of three separate experiments with similar results. *P <0.05, **P <0.01, as compared with the group without ABL-N treatment.

Bcl-2 family of proteins, including Bcl-2 and Bcl-2-related family members such as Bcl-xL, Bad and Bax, plays an important role in the regulation of apoptosis [27,28]. Thus, we evaluated the effects of ABL-N on the expression of anti-apoptotic protein Bcl-2 and the pro-apoptotic proteins Bax and Bad in breast cancer cells. Figure 4a showed a marked increase in the level of Bax and Bad, which started at six hours and peaked at 24 hours of treatment with ABL-N in MDA-MB-231 cells. In contrast, reduced Bcl-2 protein appeared later at 12 hours. Then, the ratio of Bax and Bcl-2, which is the determining factor for the induction of apoptosis [29], was measured by a densitometric analysis of the bands. As shown in Figure 4b, ABL-N treatment resulted in a time-dependent increase in Bax/Bcl-2 ratio in MDA-MB-231 cells that favors apoptosis.

### ABL-N induces the activation of JNK and p38 signaling in breast cancer cells

**Figure 5:**
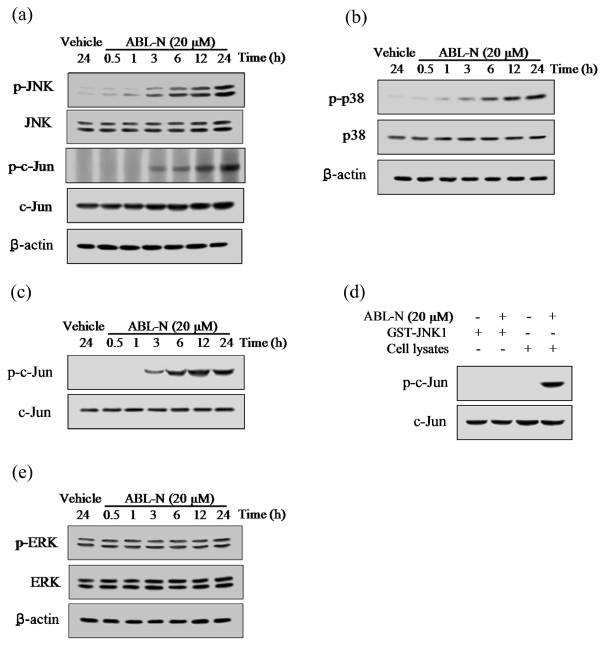
**Time course of MAP kinase activation by ABL-N**. MDA-MB-231 cells were treated with vehicle (0.5% ethanol) or 20 μM of ABL-N and harvested at the indicated times. **(a) **JNK; **(b) **p38; and **(e) **ERK activation was determined by Western blot analysis using antibodies that recognize the phosphorylated form of the respective active MAP kinase. **(c) **JNK activity was determined by an *in vitro *kinase assay as described in Materials and methods. Phosphorylation of c-Jun, which represents the intrinsic activity of JNK, was visualized by Western blotting. **(d) **ABL-N (20 μM) and GST-JNK1 fusion proteins (1 μg) were incubated for 12 hours or cells were treated with ABL-N (20 μM) for 12 hours and lysates were prepared, then JNK activity was measured as described above. Phosphorylation of c-Jun was visualized by Western blotting. The same blot was stripped and reprobed with a c-Jun antibody to monitor equal loading of proteins. Data are representative of three independent experiments.

Activation of mitogen-activated protein (MAP) kinase is involved in many aspects of the control of cellular proliferation and apoptosis in response to a variety of extracellular stimulus [30-32]. We therefore examined the effects of ABL-N on the activation of several MAP kinase pathways. The cell lysates from ABL-N-treated MDA-MB-231 cells were subjected to Western blot analysis using antibodies that specifically recognize the phosphorylated forms of JNK, p38 and ERK. As shown in Figure 5a and 5b, ABL-N treatment induced activation of JNK and p38 in a time-dependent manner.

The activation of JNK by ABL-N was further confirmed by the analysis of phosphorylation of its downstream substrate c-Jun (Figure 5a). It showed that c-Jun was phosphorylated following ABL-N treatment, which occurred over the same sustained period as JNK activation. However, no evident JNK and c-Jun phosphorylation could be observed within three hours after ABL-N treatment. Thus, JNK activation induced by ABL-N is a delayed and sustained, but not an early and transient, process. To gain further insight into the mechanism by which ABL-N treatment affects JNK, we assayed JNK activity in ABL-N-treated cells as well as the direct effect of ABL-N on GST-JNK1 fusion proteins activity. As depicted in Figure 5c, JNK activity began to increase after treatment with 20 μM ABL-N for three hours and maximum activation was achieved 12 hours after treatment. In addition, using GST-JNK1 fusion proteins, we found that the JNK activity was unaffected by the presence of ABL-N (Figure 5d), indicating ABL-N activated JNK indirectly by activating signaling molecules located upstream in the JNK cascades. On the contrary, ERK was revealed to be constitutively activated in the cells and no significant change of ERK expression and phosphorylation was observed after ABL-N treatment (Figure 5e).

### Suppression of JNK antagonized ABL-N-induced caspase-3 activity and PARP cleavage

**Figure 6:**
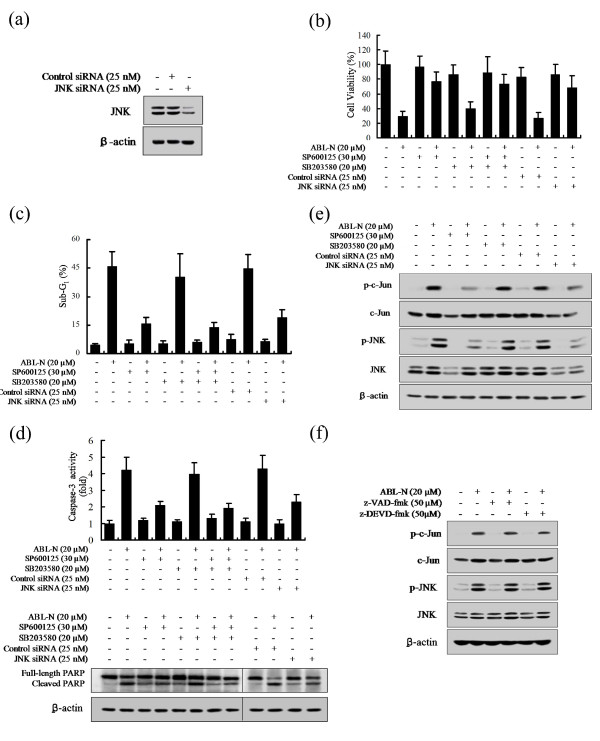
**Role of JNK in ABL-N-induced apoptosis**. MDA-MB-231 cells were incubated with ABL-N (20 μM) and either SB203580 (20 μM) or SP600125 (30 μM), or a combination of both for 24 hours. **(a) **MDA-MB-231 cells were transfected with JNK siRNA (25 nM) or control siRNA (25 nM) for 48 hours, and cell lysates were subjected to Western blot with antibodies to JNK. **(b) **ABL-N-induced cell death was abrogated by inhibition of JNK using MTT assay. **(c) **Sub G_1 _DNA content was analyzed by flow cytometry. **(d) **Caspase-3 activity was determined by Caspase-Glo assay and the cleavage of PARP was analyzed via Western blotting. Data are expressed as the means ± SE of three independent experiments. **(e) **Effect of MAP kinase inhibitors on ABL-N-induced JNK activation. **(f) **Effect of caspase inhibitors on JNK activation. Cells pretreated with or without z-VAD-fmk (50 μM) or z-DEVD-fmk (50 μM, one hour) were further incubated with vehicle or 20 μM ABL-N for 24 hours. Data are representative of three independent experiments.

To determine the role of the activation of JNK and p38 in ABL-N-induced apoptosis, MDA-MB-231 cells were treated with the JNK inhibitor SP600125 or the p38 inhibitor SB203580 and their effects on cell apoptosis were examined. Additionally, we transfected MDA-MB-231 cells with JNK siRNA or with a control siRNA for 48 hours. Transfection of JNK siRNA markedly reduced the expression of JNK protein compared with that in cells transfected with the control siRNA (Figure 6a). As shown in Figure 6b, reduction of cell viability by ABL-N was effectively abolished by SP600125, but SB203580 only had a slight effect on the decreased viability by ABL-N. The cells transfected with the JNK siRNA also blocked ABL-N-induced loss of cell viability. These results suggested that the activation of JNK signaling is responsible for the ABL-N-induced apoptosis. Similar results were further confirmed by flow cytometic analysis to determine the sub-G_1 _DNA contents (Figure 6c). Similarly, JNK inhibition by its specific inhibitor or siRNA could effectively antagonize ABL-N-induced caspase-3 activity and PARP cleavage (Figure 6d). Moreover, the stimulatory effect of ABL-N on c-Jun phosphorylation and JNK phosphorylation was not altered by SB203580, but significantly reduced by SP600125 (Figure 6e). These results suggested that activation of JNK, but not p38, was important for ABL-N-induced apoptosis.

Furthermore, to address the possible role of caspase cleavage in the activation of JNK pathway in ABL-N-induced apoptosis, we observed the effect of caspase inhibitors on JNK activation by ABL-N. The results indicated that z-VAD-fmk (50 μM) or z-DEVD-fmk (50 μM) pretreatment had no effect on c-Jun and JNK activation induced by ABL-N (Figure 6f), further suggesting that caspases were downstream targets of JNK signaling in response to ABL-N in breast cancer cells.

### ABL-N inhibits the growth of human breast cancer xenografts

**Figure 7:**
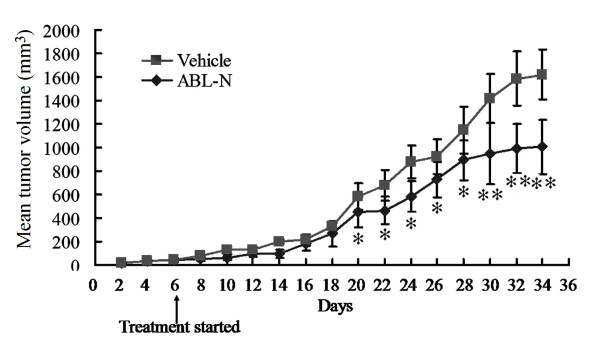
**The effect of ABL-N on the growth of MDA-MB-231 xenografts**. Female nude mice bearing MDA-MB-231 tumor xenografts were treated with ABL-N (15 mg/kg), or vehicle (10% DMSO, 10% ethanol in water), and tumors were measured with calipers on alternate days. Points = mean tumor volume in each experimental group containing six mice; bars = SD; *, *P *< 0.05; **, *P *< 0.01, compared with vehicle treated group.

Because ABL-N treatment showed the effective growth inhibition in cultured breast cancer cells, we subsequently carried out *in vivo *study using MDA-MB-231-derived cancer xenografts in nude mice. As shown in Figure 7, the i.p. treatment with ABL-N (15 mg/kg) caused a significant inhibition of tumor growth as early as 20 days after treatment and persisted after 34 days. Furthermore, animals showed no body weight loss, decreased activity, or anorexia (data not shown). This initial *in vivo *experiment suggested that ABL-N might be an effective anticancer agent at dosages that induced negligible toxic effects.

## Discussion

Induction of apoptosis in malignant cells is a critical property of chemopreventive agents. ABL, which has been shown to be potently anti-tumorigenic, has pro-apoptotic features in many carcinoma cell types [17-19]. We modified ABL to improve potency and pharmacologic characteristics, and obtained a highly active derivative ABL-N, which showed exceptional anti-proliferative activity against several human cancer cell types. The differences in biological activity of ABL and ABL-N could be attributed to the difference in their structures (Figure 1a). The increased activity of ABL-N could be, most likely, attributed to (S)-2-(6-methoxynaphthalen-2-yl)propionyl- side chain of ABL-N, which is substituted with hydroxyl group at C-6 in ABL. The purpose of this study was to investigate the possible role of caspase activation and JNK signaling in ABL-N-induced apoptosis in ER-positive and ER-negative breast cancer cells.

Apoptosis is governed by a complex network of anti-apoptotic and pro-apoptotic effector molecules. Among proteins implicated in control of apoptosis, members of the Bcl-2 family have been demonstrated to be associated with the mitochondrial membrane integrity [33]. They can activate or inhibit the release of downstream factors such as cytochrome *c *which leads to the activation of caspase-3 and PARP in the execution of apoptosis [34]. Bax exerts pro-apoptotic activity by translocation from the cytosol to the mitochondria, where it induces cytochrome *c *release, while Bcl-2 exerts its anti-apoptotic activity, at least in part, by inhibiting the translocation of Bax to the mitochondria [35,36]. Notably, the ratio of pro- and anti-apoptotic protein expression, such as Bax/Bcl-2, is critical for the induction of apoptosis, and it decides the susceptibility of cells to undergo apoptosis [29]. In the present study, we showed that treatment of the MDA-MB-231 cells with ABL-N resulted in significant decrease in the Bcl-2 protein and increase in the Bax protein, thus shifting the Bax/Bcl-2 ratio in favor of apoptosis.

Caspases are known to act as key mediators of apoptosis and to contribute to the apoptotic morphology through the cleavage of various cellular substrates. Caspase-3 is an executioner member that can cleave specific substrates such as PARP [37,38]. Here, the results that caspase-3 activation was accompanied by PARP cleavage in MDA-MB-231 and MDA-MB-468 ER-negative cells indicated that caspase-3 might play a key role as an important executioner in ABL-N-induced apoptosis in these cell lines. It was further substantiated by the data that caspase-3 inhibitor z-DEVD-fmk suppressed cell apoptosis induced by ABL-N. However, the absence of caspase-3 activation in MCF-7 ER-positive cells [39] raised the possibility that ABL-N might induce apoptosis in MCF-7 cells through caspase-3-independent pathway. It is demonstrated that curcumin and tributyrin could induce apoptosis but independent of caspase-3 activation in MCF-7 cells [40,41]. Therefore, other caspases might take part in ABL-N-induced apoptosis in MCF-7 cells. This was supported by our results that the pan-caspase inhibitor z-VAD-fmk could abrogate ABL-N-induced nucleosome fragmentation and the appearance of sub-G_1 _cells. Our study further found that ABL-N induced significant activation of caspase-8 in the breast cancer cells, suggesting that ABL-N may activate the death receptor pathway and induce the expression of death receptors or death ligands such as tumor necrosis factor-α and Fas ligand [42]. Thus, death receptor- or mitochondria-mediated activation of the caspase may be a potential mechanism underlying ABL-N-induced apoptosis in breast cancer cells. Recently, it was reported that another factor contributing to the activation of caspase is related to the inhibitors of apoptosis proteins (IAPs), which have been revealed to inhibit apoptosis due to their function as direct inhibitors of caspases [43,44]. Many anticancer agents such as curcumin [45], epigallocatechin [46] and esculetin [47], have been shown to interfere with IAPs and increase apoptosis rate. Consequently, we can hypothesize that ABL-N possibly invokes similar pathways and further study is necessary to investigate the precise mechanisms responsible.

It is reported that the JNK pathway is very important in cell apoptosis induced by various cytotoxic compounds [30,46,47]. Studies reported here showed that ABL-N induced activation of JNK beginning one hour after ABL-N treatment and produced a sustained elevation for at least 24 hours in MDA-MB-231 cells, which is much earlier than the activation of caspase and the apoptosis. Moreover, the JNK activation correlated with the increased phosphorylated c-Jun, which appeared to play a significant role in ABL-N-induced apoptosis and to correlate with the activation of caspase. ABL-N could also activate JNK in cells as monitored by JNK activity assay. Several JNK isoforms, including JNK2 and JNK3, had kinase activity in the absence of any activating upstream kinase, which showed autophosphorylation activity resulting in constitutive activation [48]. Therefore, we use recombinant JNK1 proteins to determine the direct effects of ABL-N on JNK. However, ABL-N did not activate GST-JNK1 fusion proteins *in vitro *kinase activity assay, indicating ABL-N might act on JNK by the upstream activators. Many studies have reported that activation of JNK by its upstream kinases or molecules is critical for its function. A number of factors, such as MAP kinase kinase 4 (MKK4) [49], epidermal growth factor receptor (EGFR) [50], insulin growth factor receptor (IGFR) [51] and spleen tyrosine kinase (SYK) [52], have been implicated in the activation of JNK. Thus, further studies will be required to determine how ABL-N acts upstream of JNK. In addition, our data showed that neither of the caspase inhibitors prevented ABL-N-induced JNK activation, while JNK-specific inhibitor or JNK siRNA, at least partially inhibited ABL-N-induced apoptosis, indicating that JNK is upstream of caspases in ABL-N-initiated apoptosis. Therefore, the results showed that both the caspases and JNK pathway were necessary to ABL-N-induced apoptosis in breast cancer cells because interfering with either pathway could attenuate apoptosis. Specifically, it was important to note that interference with JNK using the specific inhibitor or siRNA did not result in total abolition of ABL-N-induced cell death, as shown by MTT and flow cytometry assays. This may contribute to partial inhibition of JNK by SP600125 or siRNA, since JNK inhibition led to significant but not total reduction in phosphorylation of c-Jun. Thus, it is likely that JNK-independent mechanisms may participate in ABL-N-induced apoptosis.

## Conclusions

In summary, our studies suggest for the first time that ABL-N significantly induces apoptosis in breast cancer cells. This induction is associated with the activation of caspases and JNK signaling pathways. Moreover, ABL-N treatment caused a significant inhibition of tumor growth *in vivo*. Therefore, it is thought that ABL-N might be a potential drug for use in breast cancer prevention and intervention.

## Abbreviations

ELISA: enzyme-linked immunosorbent assay; ER: estrogen receptor; ERK: extracellular signal-regulated kinase; IAP: inhibitors of apoptosis protein; JNK: c-Jun NH_2_-terminal kinase; MAP kinase: mitogen-activated protein kinase; MTT: 3-(4,5-dimethylthiazol-2-yl)-2,5-diphenyltetrazoliumbromide; PARP: poly (ADP-ribose) polymerase; p38: p38 MAP kinase; PBS: phosphate-buffered saline; siRNA: small interfering RNA.

## Competing interests

The authors declare that they have no competing interests.

## Authors' contributions

BL designed the study, carried out many of the experiments, and drafted the manuscript. RHS, JJW and YPZ participated in the design of the study and data interpretation. MH, DQZ and JKW led the conception and design of the study and the revisions to the manuscript and supervised this research project. All authors read and approved the final manuscript.

## References

[B1] Mellstedt H Cancer initiatives in developing countries Ann Oncol 2006 17 viii24 viii31 10.1093/annonc/mdl984 16801336

[B2] Althuis MD Dozier JM Anderson WF Devesa SS Brinton LA Global trends in breast cancer incidence and mortality 1973-1997 Int J Epidemiol 2005 34 405 412 10.1093/ije/dyh414 15737977

[B3] Wu CC Chan ML Chen WY Tsai CY Chang FR Wu YC Pristimerin induces caspase-dependent apoptosis in MDA-MB-231 cells via direct effects on mitochondria Mol Cancer Ther 2005 4 1277 1285 10.1158/1535-7163.MCT-05-0027 16093444

[B4] Garcia-Carbonero R Supko JG Current perspectives on the clinical experience, pharmacology, and continued development of the camptothecins Clin Cancer Res 2002 8 641 661 11895891

[B5] Rowinsky EK Donehower RC Paclitaxel (taxol) N Engl J Med 1995 332 1004 1014 10.1056/NEJM199504133321507 7885406

[B6] Zhu JY Lavrik IN Mahlknecht U Giaisi M Proksch P Krammer PH Li-Weber M The traditional Chinese herbal compound rocaglamide preferentially induces apoptosis in leukemia cells by modulation of mitogen-activated protein kinase activities Int J Cancer 2007 121 1839 1846 10.1002/ijc.22883 17565740

[B7] Huang M Gao H Chen Y Zhu H Cai Y Zhang X Miao Z Jiang H Zhang J Shen H Lin L Lu W Ding J Chimmitecan, a novel 9-substituted camptothecin, with improved anticancer pharmacologic profiles in vitro and in vivo Clin Cancer Res 2007 13 1298 1307 10.1158/1078-0432.CCR-06-1277 17287296

[B8] Tamvakopoulos C Dimas K Sofianos ZD Hatziantoniou S Han Z Liu ZL Wyche JH Pantazis P Metabolism and anticancer activity of the curcumin analogue, dimethoxycurcumin Clin Cancer Res 2007 13 1269 1277 10.1158/1078-0432.CCR-06-1839 17317839

[B9] Liu J Zhou W Li SS Sun Z Lin B Lang YY He JY Cao X Yan T Wang L Lu J Han YH Cao Y Zhang XK Zeng JZ Modulation of orphan nuclear receptor Nur77-mediated apoptotic pathway by acetylshikonin and analogues Cancer Res 2008 68 8871 8880 18974131 10.1158/0008-5472.CAN-08-1972 PMC2679687

[B10] Wang Z Qiu J Guo TB Liu A Wang Y Li Y Zhang JZ Anti-inflammatory properties and regulatory mechanism of a novel derivative of artemisinin in experimental autoimmune encephalomyelitis J Immunol 2007 179 5958 5965 17947669 10.4049/jimmunol.179.9.5958

[B11] Zhang M Huang J Xie X Holman CD Dietary intakes of mushrooms and green tea combine to reduce the risk of breast cancer in Chinese women Int J Cancer 2009 124 1404 1408 10.1002/ijc.24047 19048616

[B12] Razis ED Fountzilas G Paclitaxel: epirubicin in metastatic breast cancer--a review Ann Oncol 2001 12 593 598 10.1023/A:1011108807105 11432615

[B13] Hainsworth JD Greco FA Etoposide: twenty years later Ann Oncol 1995 6 325 341 7619747 10.1093/oxfordjournals.annonc.a059180

[B14] Jordan MA Thrower D Wilson L Mechanism of inhibition of cell proliferation by Vinca alkaloids Cancer Res 1991 51 2212 2222 2009540

[B15] Liu B Han M Wen JK Acetylbritannilactone Inhibits neointimal hyperplasia after balloon injury of rat artery by suppressing nuclear factor-kappa B activation J Pharmacol Exp Ther 2008 324 292 298 10.1124/jpet.107.127407 17911374

[B16] Liu YP Wen JK Zheng B Zhang DQ Han M Acetylbritannilactone suppresses lipopolysaccharide-induced vascular smooth muscle cell inflammatory response Eur J Pharmacol 2007 577 28 34 10.1016/j.ejphar.2007.08.030 17915214

[B17] Bai N Lai CS He K Zhou Z Zhang L Quan Z Zhu N Zheng QY Pan MH Ho CT Sesquiterpene lactones from Inula britannica and their cytotoxic and apoptotic effects on human cancer cell lines J Nat Prod 2006 69 531 535 10.1021/np050437q 16643020

[B18] Rafi MM Bai NS Chi Tang H Rosen RT White E Perez D Dipaola RS A sesquiterpenelactone from Inula britannica induces anti-tumor effects dependent on Bcl-2 phosphorylation Anticancer Res 2005 25 313 318 15816553

[B19] Park EJ Kim J Cytotoxic sesquiterpene lactones from Inula britannica Planta Med 1998 64 752 754 10.1055/s-2006-957573 9933993

[B20] Easty GC Easty DM Monaghan P Ormerod MG Neville AM Preparation and identification of human breast epithelial cells in culture Int J Cancer 1980 26 577 584 10.1002/ijc.2910260509 6263809

[B21] Edwards PA Brooks IM Bunnage HJ Foster AV Ellison ML O'Hare MJ Clonal analysis of expression of epithelial antigens in cultures of normal human breast J Cell Sci 1986 80 91 101 3522615 10.1242/jcs.80.1.91

[B22] Adhami VM Aziz MH Mukhtar H Ahmad N Activation of prodeath Bcl-2 family proteins and mitochondrial apoptosis pathway by sanguinarine in immortalized human HaCaT keratinocytes Clin Cancer Res 2003 9 3176 3182 12912970

[B23] Han M Wen JK Zheng B Cheng Y Zhang C Serum deprivation results in redifferentiation of human umbilical vascular smooth muscle cells Am J Physiol Cell Physiol 2006 291 C50 58 10.1152/ajpcell.00524.2005 16467401

[B24] Lowe SW Lin AW Apoptosis in cancer Carcinogenesis 2000 21 485 495 10.1093/carcin/21.3.485 10688869

[B25] Jiang C Wang Z Ganther H Lu J Caspases as key executors of methyl selenium-induced apoptosis (anoikis) of DU-145 prostate cancer cells Cancer Res 2001 61 3062 3070 11306488

[B26] Kaufmann SH Desnoyers S Ottaviano Y Davidson NE Poirier GG Specific proteolytic cleavage of poly(ADP-ribose) polymerase: an early marker of chemotherapy-induced apoptosis Cancer Res 1993 53 3976 3985 8358726

[B27] Elmore S Apoptosis: a review of programmed cell death Toxicol Pathol 2007 35 495 516 17562483 10.1080/01926230701320337 PMC2117903

[B28] Willis S Day CL Hinds MG Huang DC The Bcl-2-regulated apoptotic pathway J Cell Sci 2003 116 4053 4056 10.1242/jcs.00754 12972498

[B29] Cory S Adams JM The Bcl2 family: regulators of the cellular life-or-death switch Nat Rev Cancer 2002 2 647 656 10.1038/nrc883 12209154

[B30] Wada T Penninger JM Mitogen-activated protein kinases in apoptosis regulation Oncogene 2004 23 2838 2849 10.1038/sj.onc.1207556 15077147

[B31] Kyriakis JM Avruch J Mammalian mitogen-activated protein kinase signal transduction pathways activated by stress and inflammation Physiol Rev 2001 81 807 869 11274345 10.1152/physrev.2001.81.2.807

[B32] Xia Z Dickens M Raingeaud J Davis RJ Greenberg ME Opposing effects of ERK and JNK-p38 MAP kinases on apoptosis Science 1995 270 1326 1331 10.1126/science.270.5240.1326 7481820

[B33] Adams JM Cory S The Bcl-2 protein family: arbiters of cell survival Science 1998 281 1322 1326 10.1126/science.281.5381.1322 9735050

[B34] Tafani M Schneider TG Pastorino JG Farber JL Cytochrome c-dependent activation of caspase-3 by tumor necrosis factor requires induction of the mitochondrial permeability transition Am J Pathol 2000 156 2111 2121 10854232 10.1016/S0002-9440(10)65082-1 PMC1850093

[B35] Wolter KG Hsu YT Smith CL Nechushtan A Xi XG Youle RJ Movement of Bax from the cytosol to mitochondria during apoptosis J Cell Biol 1997 139 1281 1292 9382873 10.1083/jcb.139.5.1281 PMC2140220

[B36] Wang X The expanding role of mitochondria in apoptosis Genes Dev 2001 15 2922 2933 11711427

[B37] Hunot S Flavell RA Apoptosis. Death of a monopoly? Science 2001 292 865 866 10.1126/science.1060885 11341280

[B38] Nicholson DW Thornberry NA Apoptosis. Life and death decisions Science 2003 299 214 215 10.1126/science.1081274 12522239

[B39] Janicke RU Sprengart ML Wati MR Porter AG Caspase-3 is required for DNA fragmentation and morphological changes associated with apoptosis J Biol Chem 1998 273 9357 9360 10.1074/jbc.273.16.9357 9545256

[B40] Piwocka K Bielak-Mijewska A Sikora E Curcumin induces caspase-3-independent apoptosis in human multidrug-resistant cells Ann N Y Acad Sci 2002 973 250 254 10.1111/j.1749-6632.2002.tb04643.x 12485871

[B41] Heerdt BG Houston MA Anthony GM Augenlicht LH Initiation of growth arrest and apoptosis of MCF-7 mammary carcinoma cells by tributyrin, a triglyceride analogue of the short-chain fatty acid butyrate, is associated with mitochondrial activity Cancer Res 1999 59 1584 1591 10197633

[B42] Nagata S Fas ligand-induced apoptosis Annu Rev Genet 1999 33 29 55 10.1146/annurev.genet.33.1.29 10690403

[B43] Liu B Han M Wen JK Wang L Livin/ML-IAP as a new target for cancer treatment Cancer Lett 2007 250 168 176 10.1016/j.canlet.2006.09.024 17218055

[B44] Wang L Zhang Q Liu B Han M Shan B Challenge and promise: roles for Livin in progression and therapy of cancer Mol Cancer Ther 2008 7 3661 3669 10.1158/1535-7163.MCT-08-0480 19074843

[B45] Woo JH Kim YH Choi YJ Kim DG Lee KS Bae JH Min DS Chang JS Jeong YJ Lee YH Park JW Kwon TK Molecular mechanisms of curcumin-induced cytotoxicity: induction of apoptosis through generation of reactive oxygen species, down-regulation of Bcl-XL and IAP, the release of cytochrome c and inhibition of Akt Carcinogenesis 2003 24 1199 1208 10.1093/carcin/bgg082 12807727

[B46] Qanungo S Das M Haldar S Basu A Epigallocatechin-3-gallate induces mitochondrial membrane depolarization and caspase-dependent apoptosis in pancreatic cancer cells Carcinogenesis 2005 26 958 967 10.1093/carcin/bgi040 15705601

[B47] Park C Jin CY Kim GY Choi IW Kwon TK Choi BT Lee SJ Lee WH Choi YH Induction of apoptosis by esculetin in human leukemia U937 cells through activation of JNK and ERK Toxicol Appl Pharmacol 2008 227 219 228 10.1016/j.taap.2007.10.003 18031783

[B48] Tsuiki H Tnani M Okamoto I Kenyon LC Emlet DR Holgado-Madruga M Lanham IS Joynes CJ Vo KT Wong AJ Constitutively active forms of c-Jun NH2-terminal kinase are expressed in primary glial tumors Cancer Res 2003 63 250 255 12517805

[B49] Tournier C Hess P Yang DD Xu J Turner TK Nimnual A Bar-Sagi D Jones SN Flavell RA Davis RJ Requirement of JNK for stress-induced activation of the cytochrome c-mediated death pathway Science 2000 288 870 874 10.1126/science.288.5467.870 10797012

[B50] Antonyak MA Moscatello DK Wong AJ Constitutive activation of c-Jun N-terminal kinase by a mutant epidermal growth factor receptor J Biol Chem 1998 273 2817 2822 10.1074/jbc.273.5.2817 9446590

[B51] Neumann-Haefelin E Qi W Finkbeiner E Walz G Baumeister R Hertweck M SHC-1/p52Shc targets the insulin/IGF-1 and JNK signaling pathways to modulate life span and stress response in C. elegans Genes Dev 2008 22 2721 2735 18832074 10.1101/gad.478408 PMC2559911

[B52] Kasper B Brandt E Ernst M Petersen F Neutrophil adhesion to endothelial cells induced by platelet factor 4 requires sequential activation of Ras, Syk, and JNK MAP kinases Blood 2006 107 1768 1775 10.1182/blood-2005-06-2501 16263791

